# Partial Hydrolyzed Protein as a Protein Source for Infant Feeding: Do or Don’t?

**DOI:** 10.3390/nu14091720

**Published:** 2022-04-21

**Authors:** Yvan Vandenplas, Janusz Ksiażyk, Manuel Sanchez Luna, Natalia Migacheva, Jean-Charles Picaud, Luca A. Ramenghi, Atul Singhal, Martin Wabitsch

**Affiliations:** 1 KidZ Health Castle, Vrije Universiteit Brussel (VUB), 1090 Brussel, Belgium; 2 Department of Pediatrics, Nutrition, and Metabolic Diseases, The Children’s Memorial Health Institute, 04-730 Warsaw, Poland; j.ksiazyk@ipczd.pl; 3 Neonatology Division and NICU, Hospital General Universitario “Gregorio Marañón”, Complutense University of Madrid, 28009 Madrid, Spain; msluna@salud.madrid.org; 4 Department of Pediatrics, Samara State Medical University, 443084 Samara, Russia; nbmigacheva@gmail.com; 5 Department of Neonatology, Hôpital de la Croix-Rousse, Hospices Civils de Lyon, F69677 Lyon, France; jean-charles.picaud@chu-lyon.fr; 6 CarMen Laboratory, INSERM, INRA, Claude Bernard University Lyon1, F69310 Pierre-Benite, France; 7 Department of Neuroscience, Ophthalmology, Genetics, Maternal and Child Health (DINOGMI), University of Genoa, 16147 Genoa, Italy; lucaramenghi@gaslini.org; 8 Childhood Nutrition Research Centre, PPP Department, UCL GOS Institute of Child Health, London WC1N 1EH, UK; a.singhal@ucl.ac.uk; 9 Division of Pediatric Endocrinology and Diabetes, Department of Pediatrics and Adolescent Medicine, University of Ulm, 89075 Ulm, Germany; martin.wabitsch@uniklinik-ulm.de

**Keywords:** breastfeeding, functional gastrointestinal disorder, partial hydrolysate, peptide, prevention, protein

## Abstract

Exclusive breastfeeding until the age of six months is the recommended feeding method for all infants. However, this is not possible for every infant. Therefore, a second choice of feeding, as close as possible to the gold standard, is needed. For historical reasons, this has been cow’s-milk-based feeding. This paper discusses if this second-choice feeding method should contain intact protein or partially hydrolyzed proteins. The limited data available indicates that mother’s milk is relatively rich in bioactive peptides. Whether partially hydrolyzed protein might be a protein source closer to human milk protein content than intact cow’s milk needs further research. However, more research on protein and bioactive peptides in mother’s milk should be a priority for future scientific development in this field. Results of such research will also provide an answer to the question of which option would be the best second choice for infant feeding if sufficient breast milk is not available.

## 1. Introduction

Hydrolyzed formulas for infants who are not breastfed can be of two types: partially or extensively hydrolyzed cow’s-milk-protein based. Protein hydrolysis means that proteins are broken down into smaller components or peptides. The size of the peptides determines the classification of a formula as partially hydrolyzed (most peptides with molecular weight of 3–10 kDa) or extensively hydrolyzed (peptides with molecular weight of <3 kDa) [1]. This definition is based on consensus rather than scientific evidence. Hydrolyzation results in a range or continuum of peptide sizes. However, different methods of protein hydrolysis (e.g., heat treatment, enzymatic hydrolysis) are applied by various manufacturers of infant formula. As a result, the end-products of each company differ. The peptide size of some formulas has been reported to be production dependent [2]. There is no evidence to indicate whether differences in peptide size result in different clinical outcomes, although some studies suggest that up to half of all children with a proven cow’s milk allergy (CMA) have incomplete resolution of symptoms upon treatment with an extensive whey hydrolysate [3]. Similar differences are reported for partial hydrolysates, i.e., differences in peptide size, allergenicity, and induction of tolerance [4]. Peptide size was not necessarily associated with a reduction in allergenicity in vitro, nor with oral tolerance induction in vivo, as measured by a specific IgE level [4].

In summary, hypo-allergenicity and the induction of oral tolerance are hydrolysate specific. Therefore, it is not recommended to pool data obtained from clinical trials involving different hydrolysates.

## 2. Breastfeeding

Exclusive breastfeeding up to the age of 6 months is the preferred feeding method for all infants. However, there is no evidence that breastfeeding has a preventative effect on the risk of developing allergies [5]. A crucial factor in deciding the optimal second choice of protein source for infant formula is the knowledge of the protein structure in mother’s milk. Unfortunately, this has been poorly studied. There is no evidence to suggest that intact cow’s milk protein is the protein source closest to the protein found in human milk, and there is even some data suggesting that the protein in goat’s milk, rather than cow’s milk, might be closer to that in human milk. In any case, the total protein content in human milk is much lower than in animal milk, and the casein/whey ratios differ substantially. However, these differences relate to the intact protein structure, not the size of the peptides. According to the limited data available, human milk is relatively rich in peptides because of human milk proteases [6]. Human milk contains more than 1100 unique peptides derived from the 42 milk protein hydrolysates within the mammary gland, including 306 potential bioactive peptides [7]. Milk proteases actively cleave milk proteins within the mammary gland, initiating the release of functional peptides. This means that the breastfed infant receives pre-digested proteins and numerous bioactive peptides [7]. Consequently, the protein structure in a partially hydrolyzed cow’s-milk-based whey protein infant formula may be closer to the protein structure in human milk than that in intact whey protein formulas. However, proteolysis in the milk is controlled by a balance of protease inhibitors and protease activators, so not all milk proteins are digested within the mammary gland. This seems to be important, as many bioactive milk proteins (e.g., lactoferrin, immunoglobulin) need to remain intact to function [5]. More research is needed on the protein structure in mother’s milk to improve protein similarity between first- and second-choice infant feeding.

## 3. Partial Hydrolysates

The possibility of preventing CMA and other allergies in infants is not unequivocally accepted [8,9,10]. Partial hydrolysates have been available in many countries for more than 30 years. They have been marketed in Europe as “hypo-allergenic formulae”. This terminology caused a lot of confusion, as the term “hypo-allergenic formula” is used in the US to describe a formula that is effective in the management of CMA in more than 90% of all infants, with a confidence interval of 95% [11]. In Europe, the use of “hypo-allergenic” (“HA”) was intended to indicate “reduced allergenicity”, as it was hypothesized that hydrolyzed cow’s milk protein would reduce its allergenicity. The use of the term “HA” refers to the fact that hydrolyzed protein has a reduced allergenicity in in vitro models [4] and does not focus on the clinical impact. The use of “partial” and “extensively” hydrolyzed protein removes the confusion induced by the term “hypo-allergenic”.

Qualitative changes of the peptides as a result of the method of hydrolysis may influence the potential risk of allergic disease. Consequently, the degree of hydrolysis does not always correlate with the clinical effects in trials. Since different techniques are used to hydrolyze protein, the residual allergenicity of each formula is likely to differ. Peptide size is not necessarily associated with the in vitro reduction of allergenicity, nor is it associated with oral tolerance induction in vivo, as measured by specific IgE level [2]. Infant formulas differ regarding their ability to induce T-cell proliferation and proinflammatory cytokine secretion, which could also explain the different outcomes obtained in clinical studies using infant formulas [12]. However, it should also be recognized that differences demonstrated in peptide length and structure do not necessarily imply a difference in clinical outcome.

Data from in vitro and animal studies suggest that partially hydrolyzed protein has a reduced allergenicity and increases oral tolerance [13,14,15]. Animal studies suggest that a specific partial hydrolysate decreases the risk of atopic dermatitis by improving the skin barrier [15]. However, the etiology of allergic disease, including CMA, is multifactorial, with the genetic background, environmental factors, and contact with the offending allergen all contributing to the risk of allergy. The fact that many, if not all, confounding variables can be controlled in in vitro conditions or animal studies, which is not the case in real-life clinical settings, may explain the contradictory data.

Feeding infants hydrolyzed formulas has not convincingly been shown to reduce the prevalence of allergic disease and, specifically, CMA. According to a systematic review and meta-analysis (in which all studies are grouped together independent of formula or study design), there is no evidence that partial or extensive hydrolysates reduce the risk of allergic or immune-mediated disease [9]. However, some individual studies show a different outcome.

The German Infant Nutritional Intervention (GINI) study was one of the first to report evidence for a role of a specific partially hydrolyzed whey protein in the short- and long-term prevention of allergic manifestations, mainly atopic eczema, in infants with a positive family history of atopic disease [16]. The most recent follow-up data of the GINI study also suggest a reduced risk of asthma after puberty (reduced period prevalence between 16 and 20 years) [17]. Furthermore, the outcome of the GINI study illustrates the specificity of hydrolysates, as an extensive casein hydrolysate and a partially hydrolyzed whey protein showed a significant and clinically relevant reduction in eczema risk in at-risk infants, but the extensively hydrolyzed whey protein formula did not [16,17].

Current guidelines and recommendations, which are based on defined search criteria, remain neutral and do not make recommendations for or against the use of partial hydrolysates in the prevention of allergies. For example, the European Academy of Allergy and Clinical Immunology (EAACI) issued no recommendations for or against using partially or extensively hydrolyzed formula to prevent IgE mediated food allergies in infants and young children [10]. The EAACI also recommended against feeding infants intact cow’s milk protein during the first week of life [10]. Another analysis of studies on different protein hydrolysates has led to the conclusion that their role in preventing allergies is small. However, this analysis also pooled the results from different protein hydrolysates and, therefore, does not allow an evaluation of the individual hydrolysates [18]. A meta-analysis that focuses exclusively on only one type of hydrolysate showed that this specific hydrolysate reduced the risk of eczema among children at high risk for allergies, albeit not at all time points [19]. Therefore, the recommendation of the updated guidelines on Allergy Prevention DGKJ/GPA 20/21 for at-risk infants is to consider whether an infant formula with efficacy demonstrated in allergy prevention studies is available until complementary feeding is introduced [20]. Recent guidelines do no longer recommend considering a family history of atopic disease as a risk factor [21,22].

Currently, only one observational study has suggested a possible negative outcome regarding allergenicity with a partial hydrolysate [23]. This study analyzed data from a French longitudinal birth cohort study. All children who received a HA formula based on a partially hydrolyzed protein at 2 months were consolidated into one single HA group (N = 251) and compared with children who received a cow’s milk formula at 2 months (N = 7149). Apart from the extreme imbalance of the groups, the study does not allow for any conclusions concerning the effect of a specific HA formula, as partially hydrolyzed protein HA formulas from different manufactures were used. As previously mentioned, the properties of a hydrolysate are not only determined by the degree of hydrolysis or protein source, but, crucially, are dependent on the hydrolysis process itself, which varies between manufacturers [14]. Therefore, combining data from studies using different hydrolysates is likely to be scientifically inappropriate [24].

Unfortunately, trials comparing different partial hydrolysates have not been performed.

All reviews suggest that partially hydrolyzed formulas are safe, well-tolerated, and lead to appropriate infant growth. Since formulas containing hydrolyzed proteins may be produced from any suitable protein source and by different enzymatic or chemical means, the EFSA emphasizes that the safety and suitability of each specific formula containing protein hydrolysates must be established by clinical studies [25].

Partial hydrolysates are more easily digested than intact proteins [26]. An accelerated transit time in preterm infants fed partially hydrolyzed formulas, compared to intact proteins, was demonstrated in [27]. Numerous studies suggest a benefit of partially hydrolyzed formulas in managing infantile colic, regurgitation, and constipation [28]. Unfortunately, the partial hydrolyzation of the protein is only one of several changes in formula composition in all these studies. Other changes include reduced lactose, change in lipid content, and the addition of a thickening agent, and, therefore, it is impossible to pinpoint the hydrolysate as the single influential factor.

## 4. Conclusions

The risks and benefits of choosing a partially hydrolyzed formula in non-exclusively breastfed infants should be discussed between the health care professional and the infant’s caregivers. The current stage of knowledge leads to a philosophical discussion than to an evidence-guided information. While there is a high degree of certainty that partially hydrolyzed formulas are safe, substantial proof of their benefit has not been demonstrated. Regarding allergy prevention, studies showed either no benefit or some benefit. Most studies show benefits regarding the management and prevention of functional gastrointestinal disorders, although the hydrolyzed protein was always only one of the multiple changes introduced to the formula. A conservative, evidence-based analysis would conclude that there is insufficient evidence for an active recommendation. A more positive interpretation would be that, while there is no evidence to state that there is a benefit, the possibility of some benefit cannot be ruled out. Although the knowledge that a partial hydrolysate might be closer to the protein composition in human milk than intact cow’s milk protein, and that in vitro and animal studies strongly indicate benefit might be decisive in this approach, today there is insufficient evidence to recommend their universal use. Last, but not least, clinical trials with different hydrolysates are needed.

## Data Availability

Not applicable.

## References

[1] Nutten S. (2016). Proteins, peptides and amino acids: Role in infant nutrition. Nestle Nutr. Inst. Workshop Ser..

[2] Nutten S., Schuh S., Dutter T., Heine R.G., Kuslys M. (2020). Design, quality, safety and efficacy of extensively hydrolysed formula for management of cow’s milk protein allergy: What are the challenges?. Adv. Food Nutr. Res..

[3] Petrus N.C., Schoemaker A.F., van Hoek M.W., Jansen L., Jansen-van der Weide M.C., van Aalderen W.M., Sprikkelman A.B. (2015). Remaining symptoms in half the children treated for milk allergy. Eur J. Pediatr..

[4] Bourdeau T., Affolter M., Dupuis L., Panchaud A., Lahrichi S., Merminod L., Martin-Paschoud C., Adams R., Nutten S., Blanchard C. (2021). Peptide characterization and functional stability of a partially hydrolyzed whey-based formula over time. Nutrients.

[5] Victora C.G., Bahl R., Barros A.J., França G.V., Horton S., Krasevec J., Murch S., Sankar M.J., Walker N., Rollins N.C. (2016). Breastfeeding in the 21st century: Epidemiology, mechanisms, and lifelong effect. Lancet.

[6] Dallas D.C., Murray N.M., Gan J. (2015). Proteolytic system in milk: Perspectives on the evolutionary function within the mammary gland and the infant. J. Mammary Gland. Biol. Neoplasia.

[7] Nielsen S.D., Beverly R.L., Dallas D.C. (2017). Milk proteins are predigested within the human mammary gland. J. Mammary Gland Biol. Neoplasia.

[8] Greer F.R., Sicherer S.H., Burks W.A., Abrams S.A., Fuchs G.J., Kim J.H. (2019). The effects of early nutritional interventions on the development of atopic disease in infants and children: The role of maternal dietary restriction, breastfeeding, hydrolyzed formulas, and timing of introduction of allergenic complementary foods. Pediatrics.

[9] Boyle R.J., Ierodiakonou D., Khan T., Chivinge J., Robinson Z., Geoghegan N., Jarrold K., Afxentiou T., Reeves T., Cunha S. (2016). Hydrolysed formula and risk of allergic or autoimmune disease: Systematic review and meta-analysis. BMJ.

[10] Halken S., Muraro A., de Silva D., Khaleva E., Angier E., Arasi S., Arshad H., Bahnson H.T., Beyer K., Boyle R. (2021). EAACI guideline: Preventing the development of food allergy in infants and young children (2020 update). Pediatric Allergy Immunol..

[11] American Academy of Pediatrics (2000). Committee on Nutrition. Hypoallergenic infant formulae. Pediatrics.

[12] Hochwallner H., Schulmeister U., Swoboda I., Focke-Tejkl M., Reininger R., Civai V., Campana R., Thalhamer J., Scheiblhofer S., Balic N. (2017). Infant milk formulas differ regarding their allergenic activity and induction of T-cell and cytokine responses. Allergy.

[13] Graversen K.B., Larsen J.M., Pedersen S.S., Sørensen L.V., Christoffersen H.F., Jacobsen L.N., Halken S., Licht T.R., Bahl M.I., Bøgh K.L. (2021). Partially hydrolysed whey has superior allergy preventive capacity compared to intact whey regardless of amoxicillin administration in brown Norway rats. Front. Immunol..

[14] Iwamoto H., Matsubara T., Okamoto T., Yoshikawa M., Matsumoto T., Kono G., Takeda Y. (2020). Epicutaneous immunogenicity of partially hydrolyzed whey protein evaluated using tape-stripped mouse model. Pediatric Allergy Immunol..

[15] Holvoet S., Nutten S., Dupuis L., Donnicola D., Bourdeau T., Hughes-Formella B., Simon D., Simon H.U., Carvalho R.S., Spergel J.M. (2021). Partially hydrolysed whey-based infant formula improves skin barrier function. Nutrients.

[16] von Berg A., Koletzko S., Filipiak-Pittroff B., Laubereau B., Grübl A., Wichmann H.E., Bauer C.P., Reinhardt D., Berdel D., German Infant Nutritional Intervention Study Group (2007). Certain hydrolyzed formulas reduce the incidence of atopic dermatitis but not that of asthma: Three-year results of the German Infant Nutritional Intervention Study. J. Allergy Clin. Immunol..

[17] Gappa M., Filipiak-Pittroff B., Libuda L., von Berg A., Koletzko S., Bauer C.P., Heinrich J., Schikowski T., Berdel D., Standl M. (2021). Long-term effects of hydrolyzed formulae on atopic diseases in the GINI study. Allergy.

[18] Osborn D.A., Sinn J.K., Jones L.J. (2018). Infant formulae containing hydrolysed protein for prevention of allergic disease. Cochrane Database Syst. Rev..

[19] Szajewska H., Horvath A. (2017). A partially hydrolyzed 100% whey formula and the risk of eczema and any allergy: An updated meta-analysis. World Allergy Organ. J..

[20] Worm M., Reese I., Ballmer-Weber B., Beyer K., Bischoff S.C., Bohle B., Brockow K., Claßen M., Fischer P.J., Hamelmann E. (2021). Update of the S2k guideline on the management of IgE-mediated food allergies. Allergol. Select.

[21] Australasian Society of Clinical Immunology and Allergy (ASCIA) ASCIA Guidelines—Infant Feeding and Allergy Prevention. https://www.allergy.org.au/hp/papers/infant-feeding-and-allergy-prevention.

[22] Turner P.J., Feeney M., Meyer R., Perkin M.R., Fox A.T. (2018). Implementing primary prevention of food allergy in infants: New BSACI guidance published. Clin. Exp. Allergy.

[23] Davisse-Paturet C., Raherison C., Adel-Patient K., Divaret-Chauveau A., Bois C., Dufourg M.N., Lioret S., Charles M.A., de Lauzon-Guillain B. (2019). Use of partially hydrolysed formula in infancy and incidence of eczema, respiratorysymptoms or food allergies in toddlers from the ELFE cohort. Pediatr. Allergy Immunol..

[24] Vandenplas Y., Meyer R., Chouraqui J.P., Dupont C., Fiocchi A., Salvatore S., Shamir R., Szajewska H., Thapar N., Venter C. (2021). The role of milk feeds and other dietary supplementary interventions in preventing allergic disease in infants: Fact or fiction?. Clin. Nutr..

[25] EFSA Panel on Dietetic Products, Nutrition and Allergies (NDA) (2014). Scientific Opinion on the essential composition of infant and follow-on formulae. EFSA J..

[26] Billeaud C., Guillet J., Sandler B. (1990). Gastric emptying in infants with or without gastro-oesophageal reflux according to the type of milk. Eur. J. Clin. Nutr..

[27] Picaud J.C., Rigo J., Normand S., Lapillonne A., Reygrobellet B., Claris O., Salle B.L. (2001). Nutritional efficacy of preterm formula with a partially hydrolyzed protein source: A randomized pilot study. J. Pediatric Gastroenterol. Nutr..

[28] Vandenplas Y., Salvatore S. (2016). Infant formula with partially hydrolyzed proteins in functional gastrointestinal disorders. Protein Neonatal Infant Nutr..

